# Kinetically Encoded Microstrain Governs Growth, Electronic Structure, and Functionality in Microwave-Synthesized Lu_2_O_3_:RE^3+^ Nanoparticles

**DOI:** 10.1021/acsomega.6c01544

**Published:** 2026-05-04

**Authors:** Felipe Ribeiro de Vasconcelos, Priscila Hasse Palharim, Caroline Helena Claudino, Hermi Felinto Brito, Juliana dos Santos de Souza, José Miranda de Carvalho

**Affiliations:** † Center for Natural and Humanities Sciences (CCNH), 74362 Federal University of ABC, Avenida dos Estados, 5001, Santo Andre, São Paulo 09210-170, Brazil; ‡ Institute of Chemistry, 28133 University of São Paulo, Av. Prof. Lineu Prestes, 748, São Paulo 05508-900, SP, Brazil

## Abstract

Nonequilibrium synthesis provides a powerful route to access metastable structural states that are inaccessible under near-equilibrium conditions, yet quantitative links between growth kinetics, defect structure, and functional response remain poorly understood. Here, we investigate the microwave-assisted synthesis of Eu^3+^- and Tb^3+^-doped Lu_2_O_3_ nanocrystals as a model system to elucidate how nonequilibrium crystallization kinetically encodes lattice microstrain and governs material properties. Time-resolved X-ray diffraction, transmission electron microscopy, and kinetic modeling reveal diffusion-controlled crystallite growth consistent with Ostwald ripening, accompanied by persistent microstrain that relaxes only partially with microwave irradiation time. Williamson–Hall analysis demonstrates that dopant chemistry systematically modulates the magnitude and evolution of microstrain, indicating a coupling between defect formation and mass transport under microwave heating. Photoluminescence spectroscopy shows that Eu^3+^ hypersensitive transitions and Eu–O charge-transfer states track microstrain directly, establishing lattice strain as an internal structural field that tunes local symmetry and electronic structure. In contrast, Tb^3+^ emission is primarily quenched by defect-mediated nonradiative processes, highlighting dopant-selective coupling to strain. These structure–optical correlations are mirrored in photocatalytic performance, where intermediate strain levels maximize activity by balancing internal electric fields and defect recombination. Together, these results demonstrate that microwave-assisted nonequilibrium synthesis enables the kinetic encoding of lattice microstrain, providing a general strategy to rationally design functional oxide nanomaterials through controlled growth pathways rather than composition alone.

## Graphical abstract

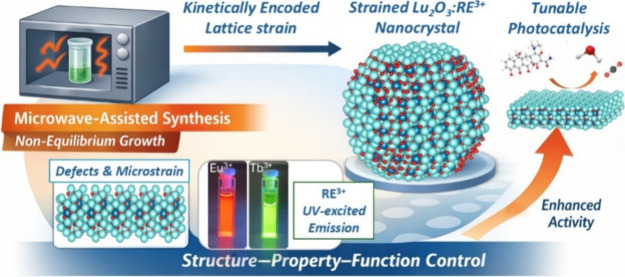

## 1 Introduction

Rare-earth sesquioxides (RE_2_O_3_) are chemically robust, wide-bandgap materials that enable a wide range of optical, electronic, and catalytic technologies.^1–4^ Despite their extensive use, how dopant chemistry influences crystallization kinetics, defect populations, and electronic behavior in rare-earth oxides—particularly under nonequilibrium synthesis conditions—remains poorly understood.^5–8^ As emphasized by Prigogine, “*non-equilibrium is the source of order*”, suggesting that deviations from equilibrium processing are not merely technical details, but fundamental variables capable of encoding structural and electronic information into crystalline materials.^9^ In this context, advanced synthesis routes that operate far from equilibrium provide a powerful platform for probing how kinetic biasing governs structure–property relationships in complex oxides.

Rare-earth elements are strategic materials for energy conversion,^10–14^ photonics, and environmental technologies, with applications ranging from permanent magnets^15,16^ and displays to optical probes and catalysts.^17–21^ In nanoparticulate form, rare-earth oxides exhibit size-, morphology-, and defect-dependent properties that are strongly coupled to their synthesis history.^22^ While redox-active oxides such as CeO_2_ have been widely studied,^23^ most heavy rare-earth sesquioxides still lack from systematic nanoparticle formation studies in nonequillibrium processes.

For photonic applications, rare-earth fluorides such as NaREF_4_ dominate due to their low phonon energies and efficient emission.^24–26^ However, their limited chemical stability and complex synthesis motivate the exploration of oxide alternatives. Rare-earth sesquioxides offer superior thermal and chemical robustness, albeit with higher phonon energies, making them ideal model systems for probing how synthesis-driven defect structures influence optical and electronic behavior.^27–30^

Among these materials, cubic Lu_2_O_3_ (space group Ia–3), is a model host because it combines several features that make it particularly suitable for probing nonequilibrium growth effects. As a well-established wide-bandgap rare-earth oxide (>5.5 eV), Lu_2_O_3_ exhibits high thermal and chemical stability, providing a robust platform for isolating synthesis-driven structural changes.^28^ Its closed-shell Lu^3+^ electronic configuration (4f^14^) suppresses host-related optical emissions, allowing the spectroscopic response of Eu^3+^ and Tb^3+^ dopants to be monitored more directly as local probes of the evolving crystal environment. In addition, Lu_2_O_3_ efficiently accommodates lanthanide dopants while preserving its simple cubic bixbyite structure, thereby facilitating analysis of lattice distortion and microstrain.^29,30^ The highly ionic character of the Lu–O lattice also offers a comparatively clean framework for discussing diffusion-mediated growth, since covalency-driven reconstruction effects are expected to be less prominent. Altogether, these attributes make Lu_2_O_3_ an ideal model system for establishing quantitative links between nonequilibrium crystallization, kinetically encoded microstrain, and functional optical and photocatalytic behavior.

Lu_2_O_3_ nanoparticles have been synthesized using a variety of solution- and gas-phase methods, including hydrothermal processing, flame spray pyrolysis, and combustion synthesis.^29,31–35^ While effective, these approaches often involve long processing times, complex reaction environments, or limited control over kinetic pathways. In contrast, microwave-assisted synthesis provides a strongly nonequilibrium environment characterized by volumetric energy deposition, rapid heating and cooling rates, and steep thermal gradients.^36–42^ Such conditions can decouple the nucleation, growth, and defect-relaxation time scales, enabling kinetic biasing of crystallization pathways that are inaccessible under equilibrium heating.

From a theoretical perspective, controlled nonequilibrium synthesis shifts the formation of materials away from free-energy minimization toward kinetic control. In diffusion-limited regimes, nanocrystal growth is often governed by Ostwald ripening, in which mass transport via dissolution–redeposition drives coarsening driven by curvature-induced chemical potential differences. In this regime, dopants can act as kinetic modifiers rather than passive electronic impurities by introducing lattice strain, modifying defect formation energies, and enhancing mass transport. However, quantitative discrimination between Ostwald ripening and alternative growth mechanisms—such as particle coalescence—remains rare for rare-earth oxides synthesized under strongly nonequilibrium conditions.^43–46^

In this work, we address this gap by employing microwave-assisted thermolysis of metal–organic Lu_1–*x*_RE_*x*_(TMA) precursors (RE = Eu, Tb; TMA: Trimesic Acid) as a model system to elucidate dopant-controlled crystallization kinetics under nonequilibrium conditions. By combining X-ray diffraction, transmission electron microscopy, microstrain analysis, and quantitative kinetic modeling, we demonstrate that crystallite growth is dominated by diffusion-controlled Ostwald ripening, with Eu^3+^ and Tb^3+^ dopants exerting distinct kinetic influences through defect-mediated mass transport. These kinetic effects are further correlated with dopant-dependent surface electronic states and band-edge energetics, as determined by photoluminescence spectroscopy, X-ray photoelectron spectroscopy, diffuse reflectance spectroscopy, and Mott–Schottky analysis. Finally, photocatalytic degradation of tetracycline under simulated solar irradiation is used as a functional probe to demonstrate that electronic and photocatalytic behavior is encoded during nonequilibrium growth, providing a direct structure–kinetics–function relationship in rare-earth sesquioxide nanocrystals. The collective results point to microstrain as a collective descriptor of local lattice distortions arising from defect populations, compositional fluctuations, and nonequilibrium relaxation fields generated during synthesis.

## 2 Experimental Part

### 2.1 Synthesis of Lu_2_O_3_:RE^3+^ Nanoparticles

#### 2.1.1 Preparation of the Metal–Organic Framework Precursors

The precursors were obtained by dissolving commercially acquired rare-earth oxides (R_2_O_3_ or Tb_4_O_7_; CSTARM, 99.99%; RE: Eu and Lu) in HCl (Labsynth, 37%) to form standard solutions of RE^3+^ chloride. For Tb_4_O_7_, a specified amount of H_2_O_2_ (Labsynth, 35%) is added dropwise to convert Tb^4+^ to Tb^3+^. The reaction products were homogenized in stoichiometric amounts according to the Lu:RE ratio (0.99:0.01). An aqueous solution of the ligand 1,3,5 sodium benzene tricarboxylate (Na_3_TMA) was prepared by neutralizing trimesic acid (H_3_TMA; Sigma-Aldrich, 99%) with NaOH (Labsynth, 99%) in a 1:3 molar ratio. Subsequently, the Na_3_TMA solution was added dropwise into the RE^3+^ aqueous solution, forming the complex Lu_0.99_RE_0.01_(TMA) (RE: Eu or Tb). The white precipitate was dried in an oven at 80 °C for 12 h and maintained in a desiccator at low pressure.

#### 2.1.2 Preparation of Lu_2_O_3_:RE^3+^ Nanoparticles via Microwave-Assisted Thermolysis

Microwave-assisted dielectric heating was performed in a hybrid mode using a susceptor.^39^ Fe_3_O_4_ was chosen as the susceptor due to the stable high-temperature heating (Figure S1). Detailed microwave assisted heating parameters are presented in Supporting Information.

In a typical synthesis, a certain amount of the precursor Lu_0.99_RE_0.01_(TMA) was placed in a 5 mL alumina crucible, which was then inserted into a larger 50 mL crucible containing a pre-established amount of a microwave susceptor (Fe_3_O_4_). The crucible was covered with an alumina lid and placed in a thermally insulating aluminosilicate block and subjected to microwave irradiation (2.45 GHz; 1600 W) for different time intervals (5, 10, 15, and 20 min). The obtained products were stored in a vacuum desiccator.

### 2.2 Characterization

The obtained nanomaterials were structurally characterized by X-ray diffraction using the powder method on a Stoe STADI-P X-ray diffractometer for polycrystals, operating in transmission mode with monochromatic CuKα1 radiation (λ = 1.5406 Å) and a Mythen 1K linear detector. Morphology, particle size, chemical composition, and electron diffraction were investigated by Transmission Electron Microscopy (TEM) using a TALOS F200X G2 transmission electron microscope (Thermo Fisher Scientific). Photoluminescence spectroscopy was conducted using an Edinburgh FS5 spectrofluorometer with a Xe lamp (150 W). The solid-state spectra were registered using the powder obtained directly from the synthesis.

Diffuse reflectance spectroscopy (DRS) was recorded with a UV–vis–NIR spectrophotometer Cary 5000 Series (Agilent Technologies) over the 200–800 nm region. Mott–Schottky plots were obtained in the dark with AC amplitudes of 10 mV and frequencies of 10, 100, and 1000 Hz. Initially, suspensions of each material at a concentration of 1 mg mL^–1^ in isopropanol were prepared and sonicated for 90 min. The resulting suspensions were dropped onto the FTO substrate and air-dried. Electrochemical measurements were conducted using a three-electrode setup connected to a potentiostat/galvanostat (μAutolab III). The Lu_2_O_3_ nanoparticle films served as working electrodes, Ag/AgCl was the reference electrode, and Pt wire acted as the counter-electrode.

### 2.3 Photocatalytic Tests

The photocatalytic performance of the synthesized materials was evaluated using tetracycline (TC) as a model pollutant. In a 35 mL quartz tube, 10 mg of catalyst was dispersed in 20 mL of a 30 mg L^–1^ TC solution. The suspension was stirred in the dark overnight to achieve adsorption–desorption equilibrium. Subsequently, the mixture was irradiated under UV–vis light from a solar simulator equipped with an A.M. 1.5G filter (100 mW cm^–2^), positioned at a fixed horizontal distance from the sample. The reaction was carried out for 120 min. During irradiation, 250 μL aliquots were withdrawn at predetermined time intervals, diluted twice, filtered, and analyzed by high-performance liquid chromatography (HPLC). TC concentration was determined using a Shimadzu LC-20 HPLC system equipped with a Prominence C18 column and a UV–vis detector (SPD-20A). The mobile phase consisted of acetonitrile (B) and 1% formic acid in water (A) with the following gradient: 15% A (0–5 min), 30% A (5–10 min), decreased to 15% A (10–12 min), and held at 15% A (12–15 min). The flow rate was 1.0 mL min^–1^, the injection volume 100 μL, and the column oven temperature 35 °C. The retention time for TC was approximately 6 min, and the detection wavelength was set at 355 nm. The limits of detection and quantification for TC were 0.04 and 0.12 mg L^–1^, respectively.

## 3 Results and Discussion

### 3.1 Structure and Morphology

The obtaining of Lu_2_O_3_:RE^3+^ (RE:Eu, or Tb) nanoparticles from TMA ligand was carried out by a microwave-assisted thermolysis of a metallic organic framework (MOF) based on trimesic acid. XRD patterns (Figure 1) of this material with an increasing microwave exposure time (5, 10, 15, and 20 min) suggest the conversion of the (Lu_0.99_RE_0.01_)­TMA system to Lu_2_O_3_:(1%)­RE^3+^,(RE:Eu or Tb), indicating that there are no crystalline intermediates detectable.

**Figure 1:**
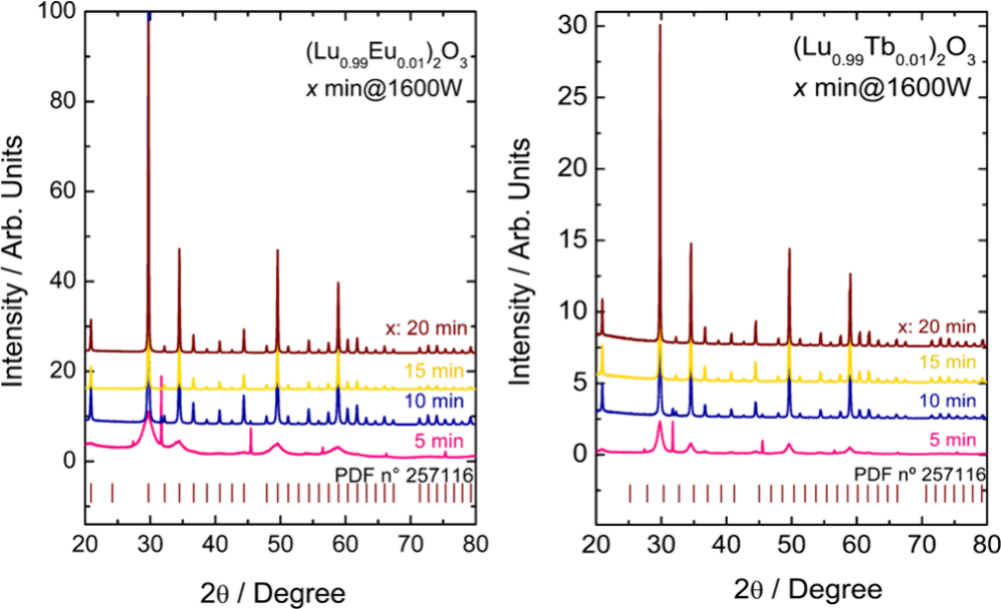
X-ray powder diffraction of the Lu_2_O_3_:RE^3+^ (RE: Eu (left), and Tb (right)) nanomaterials obtained by different microwave irradiation times (5, 10, 15, and 20 min). Standard Bragg reflection is also provided (PDF n° 257116).

It is observed that the precursor presented an amorphous broad band with only two diffraction peaks at 31° and 45° related to the (Lu_0.99_RE_0.01_)­TMA complex precursor (MOF-76).^47^ The Lu_2_O_3_:(1%)­RE^3+^ nanomaterials begin to crystallize after 5 min of microwave irradiation, evidenced by the diffraction peaks attributed to the cubic phase of Lu_2_O_3_ (space group: *Ia*3̅, PDF n° 257116).^48^ However, traces of crystalline (Lu_0.99_Tb_0.01_)­TMA complex precursor are still present after 10 min of irradiation. The diffraction peak intensities increase monotonically with increasing irradiation time from 5 to 20 min. The observed crystallization behavior is attributed to the increase in temperature of the reaction medium, which gradually degrades the organic moieties of the MOF precursor, generating the Lu_2_O_3_:RE^3+^ nanoparticles.

The evolution of the crystalline domains during synthesis provides evident insight into the fundamental mechanism governing particle growth. Crystallite sizes (Figure 2a) extracted from XRD using the Scherrer equation^49^ exhibit a monotonic increase with reaction time for both Lu_2_O_3_:RE^3+^ compositions. Although the absolute particle sizes differ slightly when comparing Lu_2_O_3_:Tb^3+^ and Lu_2_O_3_:Eu^3+^ the overall trend remains unchanged. This robustness indicates that the underlying physical process is not an artifact of data filtering but reflects an intrinsic growth mechanism.

**Figure 2:**
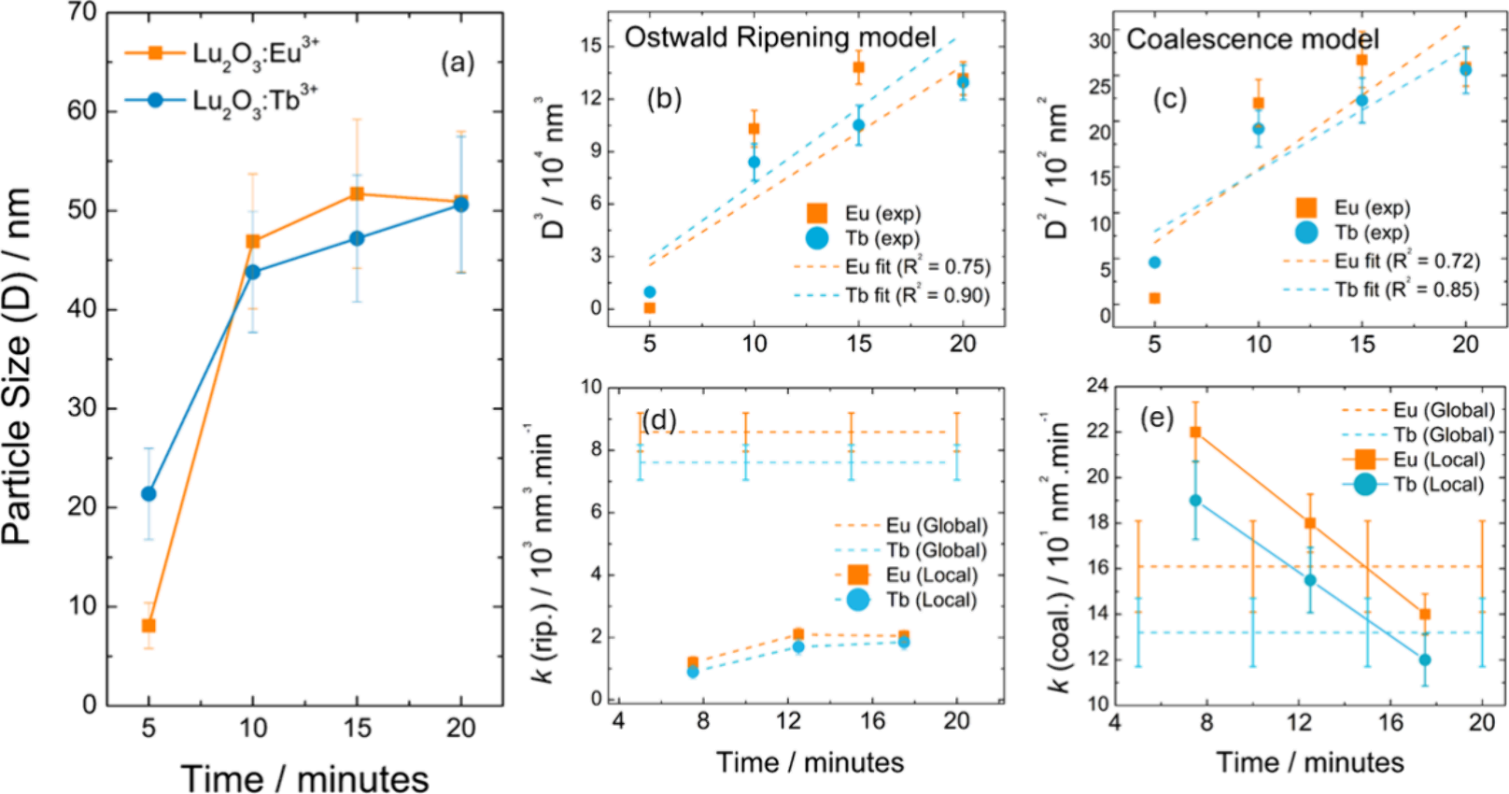
Mean particle sizes of Lu_2_O_3_:RE^3+^ (RE: Eu or Tb) materials obtained by the Scherrer method (a). Particle growth modeling for both Ostwald ripening (b), and coalescence (c) models. Global and local reaction rate constants obtained for both Ostwald ripening (d), and coalescence (e) models.

To elucidate this mechanism, the Scherrer-derived crystallite sizes were fitted to two classical kinetic models:^5,50–52^ Diffusion-controlled Ostwald ripening (1) and Collision-controlled coalescence (2).

$$D^{3}=k\cdot t+D_{0}^{3}$$ 1

$$D^{2}=k\cdot t+D_{0}^{2}$$ 2

where *D* is the particle diameter, *t* is the synthesis time, and *k* is the rate constant. *D*_0_ is the extrapolated value for the initial particle sizes. Ostwald ripening proceeds by dissolution–reprecipitation driven by curvature-induced chemical potential differences and obeys a cubic growth law *D* ^3^ ∝ *t*. Coalescence, in contrast, requires particle–particle collisions followed by fusion and is characterized by a quadratic growth law *D* ^2^ ∝ *t*.

Both Lu_2_O_3_:Tb^3+^ and Lu_2_O_3_:Eu^3+^ exhibit growth kinetics characteristic of dissolution–redeposition, where large crystallites grow at the expense of smaller ones. Fitting the particle evolution to the global zero-forced model (Figure 2b,c), the form yields a global Ostwald ripening constant (*k*_rip_(Eu) = 8.58 × 10^3^ nm^3^/min; *k*_rip_(Tb) = 7.61 × 10^3^ nm^3^/min) significantly larger than the coalescence constant (*k*_coal_(Eu) = 1.61 × 10^2^ nm^2^/min; *k*_coal_(Tb) = 1.32 × 10^2^ nm^2^/min) (Table S1). The results indicate that ripening is the dominant pathway in both systems, with the coalescence mechanism playing a secondary role. Instantaneous (local) rate constants (Figure 2d,e), obtained from interval-wise finite differences of *D* ^3^ and *D* ^2^, further emphasize this behavior: *k*_rip_ consistently exceeds *k*_coal_ by nearly 2 orders of magnitude.

Although the crystallite sizes of Eu^3+^- and Tb^3+^-doped samples may overlap within experimental uncertainty at specific synthesis times, the extracted kinetic parameters and microstrain evolution consistently indicate faster diffusion-mediated ripening dynamics in the Eu^3+^-doped system.

The relatively modest R^2^ values obtained from the linear fits reflect the nonideal, nonequilibrium nature of the growth process, such as the microwave-assisted processes, where defect-mediated fluctuations and transient structural rearrangements introduce deviations from classical ripening behavior, as commonly observed in nanocrystalline oxide systems.

The distinct crystallization behavior can be rationalized in terms of dopant-induced lattice strain and its impact on defect mobility. Eu^3+^ (ionic radius = 0.95 Å, CN = 6) exhibits a larger size mismatch relative to Lu^3+^ (0.86 Å) than Tb^3+^ (0.92 Å), leading to stronger distortions around the dopant sites.^53,54^ Such strain fields enhance the mobility of oxygen vacancies and Lu–O species, thereby increasing the dissolution–redeposition rate that drives Ostwald ripening. In contrast, the minor mismatch of Tb^3+^ yields weaker local strain, a lower concentration of mobile point defects, and consequently slower coarsening kinetics. This interpretation is consistent with the experimental observations: Eu-doped samples reach larger Scherrer crystallite sizes at equivalent synthesis times, while Tb-doped samples evolve more gradually. This dopant-sensitive ripening behavior highlights the key role of local lattice distortions and defect mobility in determining the temporal evolution of heavy rare-earth sesquioxide nanocrystals. The results emphasize that even at low concentrations (1 mol %), aliovalent or isovalent dopants can exert a strong kinetic influence on crystallization pathways through subtle modifications of the underlying energy landscape.

Williamson–Hall analysis^55^ (Table S2, and Figure S2) reveals a measurable microstrain component in Lu_2_O_3_:RE^3+^ nanoparticles at intermediate synthesis times, which decreases systematically with increasing irradiation time. Eu-doped samples consistently exhibit higher microstrain values than Tb-doped ones, particularly at early and intermediate stages, indicating a higher density of lattice distortions and defects. This enhanced microstrain correlates directly with the larger local and global Ostwald ripening rate constants observed for Eu-doped Lu_2_O_3_, supporting a defect-mediated diffusion mechanism. The local and global rate constants exhibit a similar positive trend (Figure S3), indicating that defect-induced microstrain governs diffusion-controlled crystallite growth. Notably, the progressive relaxation of microstrain coincides with the temporal decrease in local ripening rates, reinforcing the conclusion that strain-related defects facilitate mass transport during coarsening. One-way ANOVA^56^ showed the absence of statistically significant changes in the lattice parameter (Table S3), confirming that these effects arise from local lattice distortions rather than bulk structural expansion.

The morphology of the nanoparticles was examined by transmission electron microscopy (TEM). The Lu_2_O_3_:Tb^3+^ materials synthesized via microwave-assisted thermolysis exhibited a nanoparticulate morphology at all irradiation times (Figure 3a–d). After 5 min of irradiation, the samples consisted primarily of spherical nanocrystalline seeds formed during the decomposition of the Lu_0.99_Tb_0.01_(TMA) MOF precursor. Extending the irradiation time to 10 min resulted in well-defined nanospheres with an average diameter of ∼40 nm. After 15 min, polyhedral nanoparticles resembling elongated nanobricks emerged, suggesting preferential growth along the {440} family of crystallographic planes. At 20 min, pronounced aggregation of the nanostructures was observed, likely promoted by the elevated temperatures reached during microwave irradiation (∼1300 K). Even in this sintering regime, preferential growth was maintained, as evidenced by longitudinal, tetragonal-like domains interconnected into chain-like assemblies rather than forming isotropic agglomerates.

**Figure 3:**
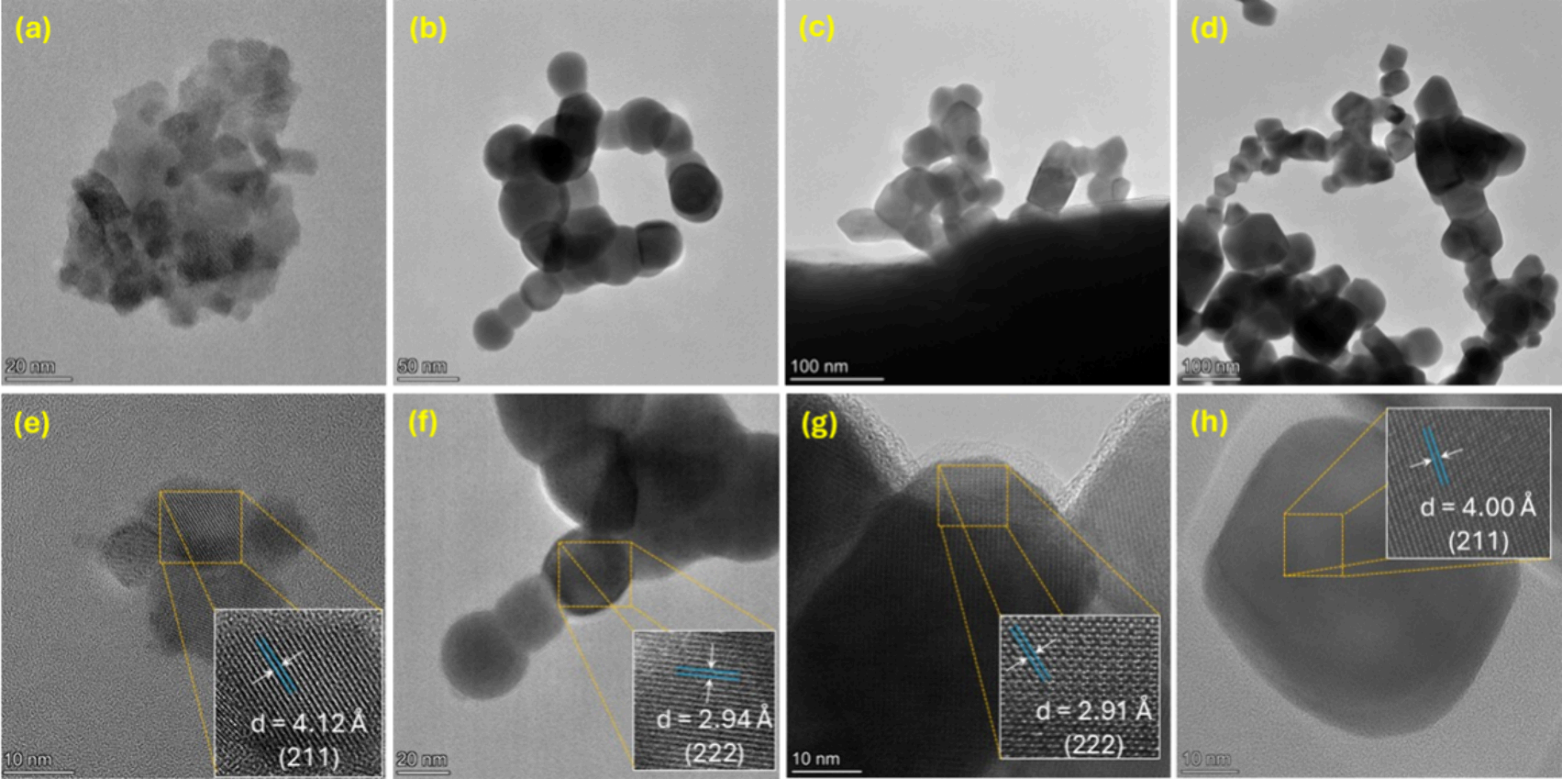
Transmission electron microscopy images showing the effect of the microwave irradiation time (5 min (a, e), 10 min (b, f), 15 min (c, g), and 20 min (d, h)) on the morphology of Lu_2_O_3_:Tb^3+^ particles. The insets show the crystallographic plane assignments obtained by reverse Fourier transform of the selected-area electron diffraction (SAED).

A similar morphological evolution was observed for the Lu_2_O_3_:Eu^3+^ samples (Figures S5 and S6), reflecting the same nucleation–growth–coarsening sequence characteristic of rapid thermal decomposition under microwave irradiation. However, subtle differences were found between the two dopants. The Eu-doped materials displayed slightly more pronounced particle coarsening and sintering, consistent with their higher local coalescence constants. TEM images showed that Eu-doped nanoparticles tended to form more continuous aggregates and exhibited an earlier onset of morphological elongation compared to their Tb counterparts. This behavior is attributed to the slightly larger ionic radius and higher defect mobility induced by Eu^3+^ substitution, which facilitates diffusion-driven coarsening at elevated temperatures. Overall, both dopants follow similar growth pathways. Still, the Eu-containing samples exhibit enhanced sintering and faster progression toward interconnected, partially fused nanostructures, in agreement with the kinetic parameters extracted from XRD analysis.

Selected-area electron diffraction (SAED) patterns acquired from particles of different sizes and morphologies revealed interplanar spacings consistent with those reported in the standard CIF file (PDF n° 257116). The SAED analysis further confirms that the individual nanoparticles are single crystals exhibiting the cubic Lu_2_O_3_ structure (space group: *Ia*3̅) (Figure 3e–h). EDS analyses (Figures S5–S8) confirm the chemical composition of the particles, showing a homogeneous dispersion of the Lu, O, RE elements throughout the samples.

The different morphologies can be attributed to the direct coupling of microwaves with the precursor, which promotes increased ionic diffusion and accelerates reaction rates, leading to a swift synthesis of the nanocrystals.^36,57,58^ Microwave-assisted thermolysis creates a strongly nonequilibrium environment in which nucleation, growth, and defect evolution occur simultaneously, enabling the kinetic encoding of microstructural features.^36,37,39,57–59^ Importantly, when the microwave process is rapidly quenched, these kinetically defined structural and morphological features can be partially or fully preserved. Consequently, the observed crystallite sizes, microstrain levels, growth kinetics, and different morphologies should be viewed as fingerprints of the microwave-driven nonequilibrium synthesis.

Particle size distribution for the increasing synthesis time was calculated using the TEM images (Figure S4). The mean particle sizes of 10 nm (5 min), 49 nm (10 min), 65 nm (15 min), and 58 nm (20 min) display an asymptotic growth trend. The slight decrease in mean size at prolonged irradiation (20 min) likely reflects additional morphological dynamics, including aggregate restructuring during TEM sample preparation.

The observed particle size distributions (Figure S4) are slightly right-skewed and well fit by log-normal distributions, in agreement with expectations for Ostwald ripening.^60^ Size-dependent growth kinetics and multiplicative stochastic variations in local dissolution–redeposition fluxes naturally lead to a log-normal-type distribution, a behavior widely reported in ripening-controlled nanocrystal systems.^51,52,61^ Thus, the experimentally measured particle-size distributions for Lu_2_O_3_:Tb^3+^ and Lu_2_O_3_:Eu^3+^ are entirely consistent with Ostwald ripening.

Taken together, the combined XRD–TEM analysis reveals a hierarchical growth mechanism (Figure 4): (i) Rapid precursor decomposition and supersaturation buildup, (ii) Nucleation and formation of defect-rich nanocrystalline seeds. (iii) Diffusion-controlled Ostwald ripening. (iv) Defect relaxation, partial coalescence, and kinetic arrest. Limited coalescence may occur due to particle–particle contact at elevated temperatures, but it remains secondary to ripening. Upon rapid quenching of the microwave process, the kinetically defined crystallite sizes, microstrain states, and morphologies are preserved, reflecting the nonequilibrium growth pathway.

**Figure 4:**
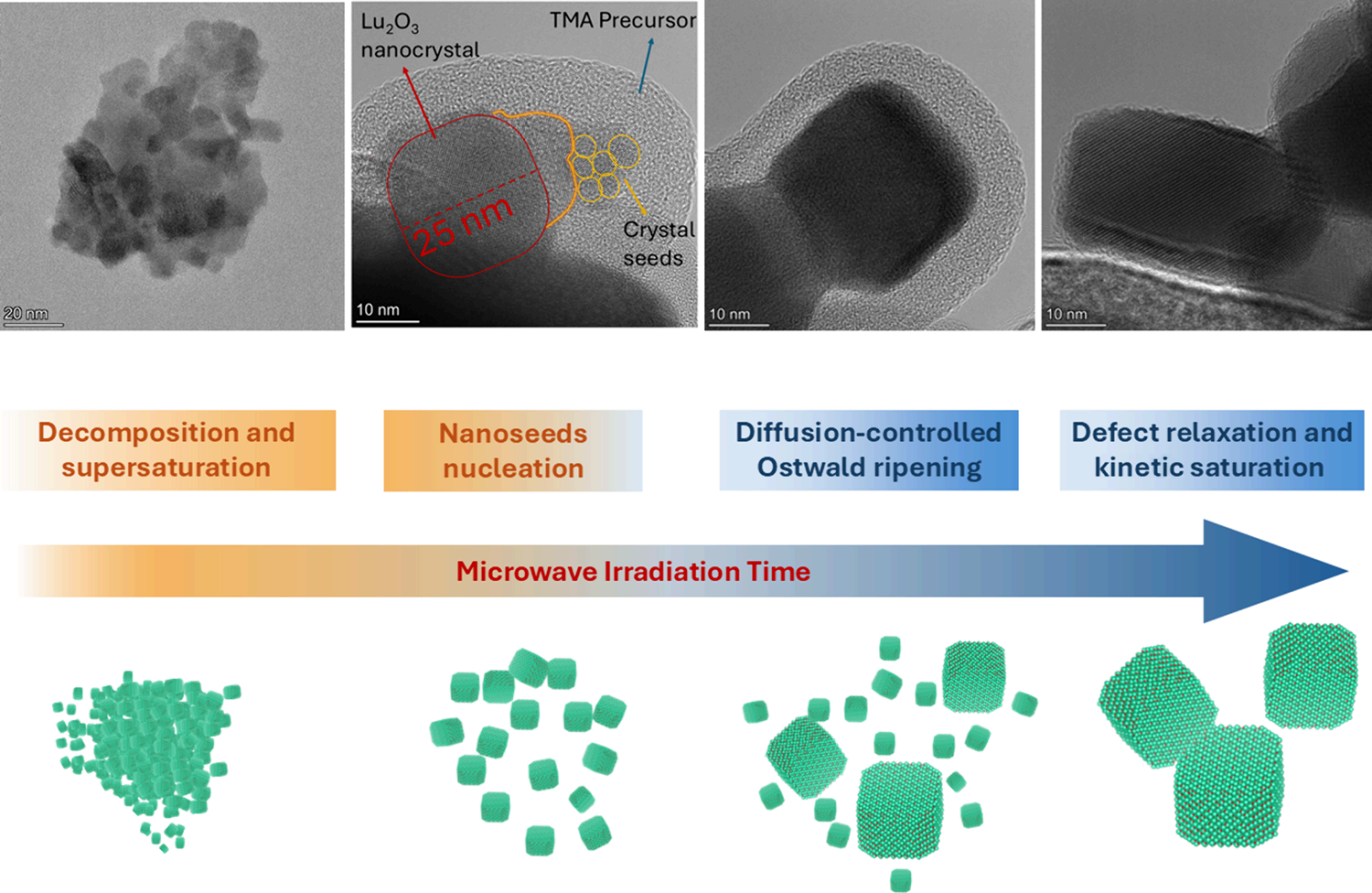
Schematic illustration of the nonequilibrium, microwave-driven formation pathway of Lu_2_O_3_:RE^3+^ nanoparticles, highlighting the transition from rapid precursor decomposition and burst nucleation to diffusion-controlled Ostwald ripening and defect-relaxation-limited growth.

### 3.2 Surface Characterization

X-ray photoelectron spectroscopy (XPS) was employed to elucidate the chemical states and surface evolution of Lu_2_O_3_:RE^3+^ materials subjected to varying microwave irradiation times (0–20 min) (Figure S9). The Lu 4d spectra display two well-defined spin–orbit components at ∼197 eV (4d_5/2_) and ∼206 eV (4d_3/2_), with an average splitting of ∼9.6 eV, consistent with the trivalent oxidation state of lutetium (Lu^3+^) in Lu_2_O_3_.^62–64^ The gradual shift of both components toward lower binding energies with increasing irradiation time suggests a reduction in surface charging effects and possible improvement in crystallinity or electronic screening within the oxide lattice.

The C 1s and O 1s regions reveal notable changes in surface chemistry induced by microwave irradiation. The C 1s spectra evolve from strong contributions at 288–289 eV, assigned to oxidized carbon species (C=O, CO_3_^2–^), toward dominant peaks at ∼285 eV, indicative of adventitious carbon.^62,65^ Simultaneously, the O 1s main peak centered at ∼531 eV, corresponding to lattice oxygen (O^2–^), becomes more pronounced relative to the higher-binding-energy component at ∼533 eV, associated with surface hydroxyls and adsorbed carbonates. These trends indicate the progressive conversion of the Lu_0.99_RE_0.01_TMA precursor to Lu_1.98_RE_0.02_O_3_ nanomaterials, accompanied by the removal of adsorbed species and carbonaceous residues upon microwave exposure, resulting in a cleaner oxide surface.^66^

In Tb-doped samples, the Tb 3d_5/2_ and 3d_3/2_ peaks at ∼1276 and ∼1241 eV, respectively, exhibit a constant spin–orbit splitting of ∼35 eV, characteristic of Tb^3+^. The invariant Δ*J* values across all irradiation conditions further confirm that neither lutetium nor terbium undergoes a change in oxidation state during processing.^67^

However, in the Eu-doped samples, both Eu^2+^ and Eu^3+^ species are observed at the material surface with energies 1125 and 1135 eV, respectively, for all microwave irradiation times. This indication may favor the charge dynamics of the material surface.^68^ The presence of Eu^2+^ species detected by XPS suggests partial reduction of Eu^3+^ under the synthesis conditions. This behavior can be rationalized by considering the nonequilibrium and defect-rich environment generated during microwave-assisted synthesis. In particular, the formation of oxygen vacancies and associated charge-compensation mechanisms may locally stabilize the Eu^2+^ oxidation state. Because XPS is intrinsically surface-sensitive, the detected Eu^2+^ species are likely enriched at or near the nanoparticle surface, where defect concentrations and nonequilibrium coordination environments are expected to be highest

### 3.3 Photoluminescence Response and Coupling to Kinetically Encoded Microstrain

Figure 5a shows the excitation spectra of the Lu_2_O_3_:Eu^3+^ nanoparticles obtained with different microwave irradiation in the spectral range of 250–500 nm, with emission centered at 611 nm corresponding to the forced-dipole hypersensitive transition of Eu^3+^ (^5^D_0_ → ^7^F_2_). A broad band with a maximum at approximately 245 nm can be observed, attributed to the O^2–^ → Eu^3+^ charge transfer (CT). The progressive red shift of the charge-transfer band from 240.1 (5 min), 241.5 (10 min), 242.7 (15 min), to 243.5 nm (20 min) with increasing synthesis time correlates directly with crystallite growth and microstrain relaxation. Smaller particles exhibit blue-shifted CT transitions due to surface-dominated, under-coordinated Eu–O environments, whereas larger crystallites display bulk-like Eu–O coordination and reduced lattice distortion.^69,70^ Narrow bands excitation from 350 and 450 nm are also present, attributed to Laporte-forbiden (Δ*l* = ± 1) localized Eu^3+^ f-f transitions.

**Figure 5:**
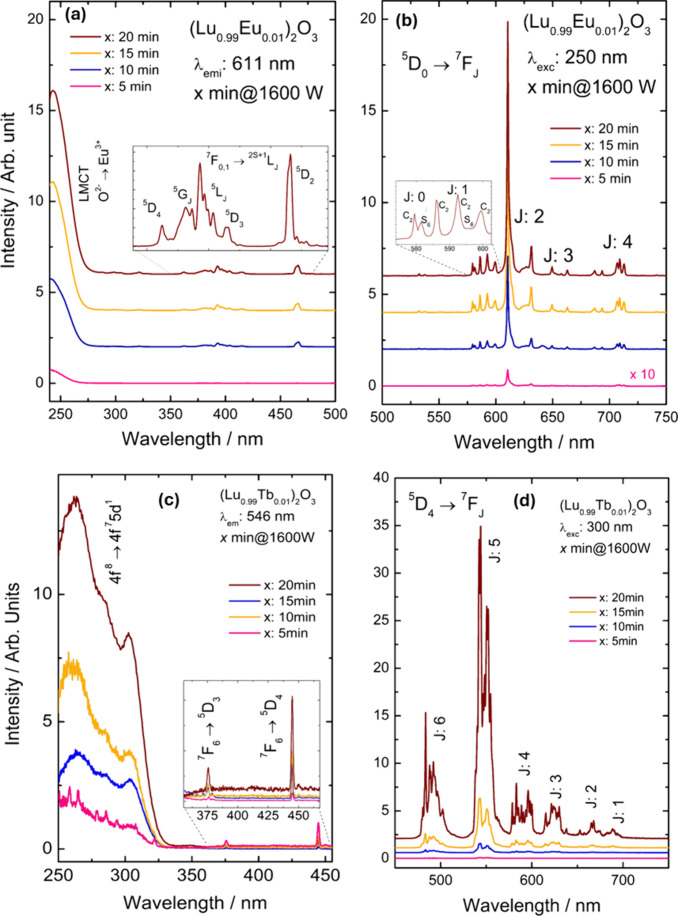
Photoluminescence excitation and emission for the Lu_2_O_3_:Eu^3+^ (a, b) and Lu_2_O_3_:Tb^3+^ (c, d) materials obtained by different microwave irradiation times.

Figure 5b shows the emission spectra of the Lu_2_O_3_:Eu^3+^ material in the spectral range of 450–750 nm, with excitation centered at 265 nm. The observed bands can be attributed to the intraconfigurational transitions of the Eu^3+^ ion (^5^D_0_ → ^7^F_J_). It is important to note that the hyperfine structures provide information about the chemical environment of the Eu^3+^ ion. In this context, the ^5^D_0_ → ^7^F_0_ transition serves as a probe of the local structure, typically showing only one peak due to the 2*J* + 1 rule. However, two peaks are observed for the ^5^D_0_ → ^7^F_0_ transitions, indicating that the Eu^3+^ ion occupies two distinct sites. Analyzing the crystallographic structure of the material, two possible Lu^3+^ sites (S_6_ and C_2_) are identified as capable of accommodating the Eu^3+^ ion.

Figure 5c shows the excitation spectrum of Lu_2_O_3_:Tb^3+^ materials in the range of 250–450 nm, with dominant emission centered at 550 nm attributed to the Tb^3+^ f–f transition (^5^D_4_ → ^7^F_5_). The broad excitation band observed between 250 and 350 nm is attributed to the allowed 4f^8^ → 4f^7^5d^1^ transition of Tb^3+^. As a Laporte-allowed transition, it provides efficient absorption within this spectral region. It is evident that microwave irradiation time increases the excitation intensity of the Tb^3+^ ion for the same reasons of the Eu^3+^-doped ones.

Figure 5d shows the emission spectrum of Lu_2_O_3_:Tb^3+^ nanoparticles in the spectral range of 400–750 nm, with excitation centered at 300 nm. For all materials, narrow emission bands attributed to the intraconfigurational f–f transitions of the Tb^3+^ ion (^5^D_4_ → ^7^F_J_; *J*: 6–0) can be observed. The bands exhibit hyperfine structure due to interaction with the crystal field, also known as spin–orbit coupling. This coupling can give rise to up to 2*J* + 1 peaks for each observed transition.

The photoluminescence properties of Lu_2_O_3_:Eu^3+^ and Lu_2_O_3_:Tb^3+^ nanocrystals synthesized under microwave irradiation were investigated to elucidate how nonequilibrium growth influences the f-f transitions. The optical response of rare-earth activators is expected to be influenced by lattice distortion and microstrain arising from the local coordination environment. Thus, the emphasis was placed on correlating spectroscopic observables with independently extracted microstrain values obtained from Williamson–Hall analysis of XRD data.

#### 3.3.1 Eu^3+^-Doped Lu_2_O_3_: Local Symmetry and Electronic Structure Probed by PL

The emission spectra of Eu^3+^-doped Lu_2_O_3_ are dominated by the characteristic ^5^D_0_ → ^7^F_J_ transitions, with the hypersensitive, forced electric-dipole ^5^D_0_ → ^7^F_2_ transition that exhibits strong coupling to the local site symmetry. The asymmetry ratio *R* = (^5^D_0_ → ^7^F_2_)/*I*(^5^D_0_ → ^7^F_1_), was extracted by spectral integration and used as a quantitative descriptor of the Eu^3+^ coordination environment. The highest asymmetry ratio (Figure 6a,b) is observed for the sample synthesized at 5 min of microwave irradiation and coincides with the largest microstrain (ε ≈ 6.0 × 10^–4^). This regime corresponds to extreme nonequilibrium growth, where rapid nucleation and limited atomic rearrangement produce highly distorted and heterogeneous Eu–O environments. As microwave irradiation time increases, ε decreases systematically, reflecting partial lattice relaxation during crystallite growth. When the analysis is restricted to the 10–20 min interval, a clear correlation emerges between decreasing ε and the evolution of *R*, indicating that Eu^3+^ can act as a spectroscopical probe of the kinetically encoded microstrain.

**Figure 6:**
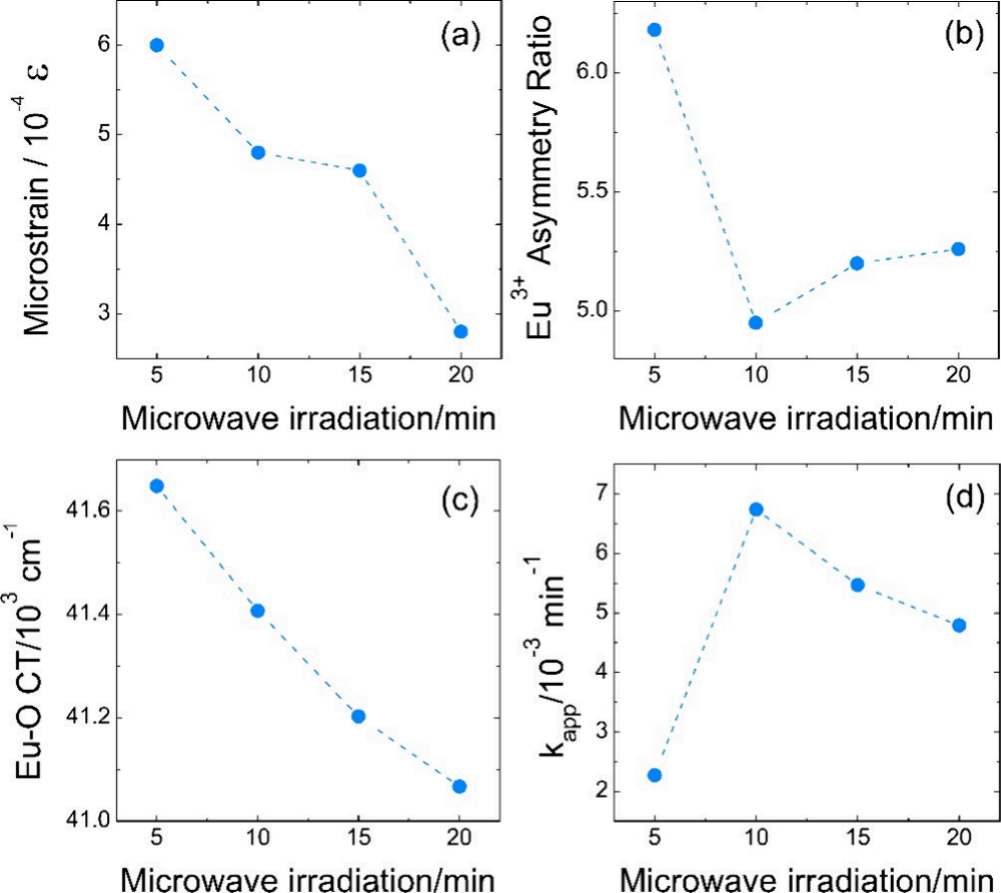
Effect of the microwave irradiation on the microstrain (a), Eu^3+^ asymmetry ratio (b), Eu–O charge transfer energy (c), and apparent constant of tetracycline degradation kinetics (d).

Importantly, the dependence of *R* on ε is nonlinear across the full-time range. While extreme strain at early times enhances site heterogeneity and maximizes *R*, further strain relaxation leads to a more coherent distortion of the Eu–O coordination. The behavior is consistent with the synthesis mechanism, which assumes a supersaturated environment with coupled nucleation and mass transport. Increasing microwave irradiation leads to the redistribution of Eu^3+^ ions between surface and near-bulk sites as crystallites coarsen and defects reorganize.

Independent confirmation of strain-mediated electronic structure modification is provided by the evolution of the Eu–O charge-transfer (CT) band (Figure 6c). The CT band exhibits systematic redshift from ∼41650 cm^–1^ (5 min) to ∼41060 cm^–1^ (20 min). When compared with the microstrain (ε), the CT energy shows a monotonic dependence, indicating progressive relaxation of the Eu–O bonding environment with decreasing lattice distortion. The CT band probes ligand–metal orbital environment and thus provides complementary evidence that microstrain modifies the electronic structure at the bonding level.

Together, the correlated evolution of asymmetry ratio and CT energy demonstrates that microstrain is not merely a byproduct of microwave synthesis, but a physically meaningful variable that tunes both local symmetry and Eu–O electronic coupling. It is reflected even in the photocatalytic constant rates (Figure 6d), which show a strong negative correlation with both microstrain and the asymmetry ratio.

#### 3.3.2 Tb^3+^-Doped Lu_2_O_3_: Defect-Mediated Quenching

In contrast to Eu^3+^, the emission of Tb^3+^-doped Lu_2_O_3_ is dominated by the ^5^D_4_ → ^7^F_J_ transitions, which are primarily magnetic-dipole in nature and therefore weakly sensitive to local symmetry. As a result, asymmetry-based metrics analogous to those used for Eu^3+^ are not applicable. Instead, the integrated Tb^3+^ emission intensity was analyzed as a function of microstrain.

The Tb^3+^ PL intensity exhibits pronounced variation with irradiation time (Figure 5d), increasing substantially as ε decreases from ∼4.5 × 10^–4^ (5 min) to ∼2.5 × 10^–4^ (20 min). This is attributed to the suppression of nonradiative recombination pathways associated with strain-induced defects and surface disorders.

Notably, no clear monotonic correlation is observed between ε and the apparent photocatalytic rate constants for Tb^3+^-doped samples. This contrasts sharply with the Eu^3+^ system and underscores the selective coupling between lattice strain and different optical and functional probes.

#### 3.3.3 Implications for Nonequilibrium Design of Functional Oxides

The PL analysis establishes that microwave-assisted synthesis enables the kinetic encoding of microstrain, which persists over experimentally relevant time scales and selectively governs optical response depending on dopant chemistry. Eu^3+^-doped Lu_2_O_3_ exemplifies how strain can be exploited to tune local symmetry and electronic structure, while Tb^3+^-doped materials highlight the role of strain in defect suppression and emission efficiency.

This dopant-selective behavior demonstrates that microstrain is not a universal control parameter affecting all observables equally, but rather an internal variable whose influence depends on the coupling strength between lattice distortion and the relevant electronic transitions.

These findings demonstrate that nonequilibrium synthesis pathways offer access to metastable structural states that cannot be achieved under near-equilibrium conditions, providing a powerful strategy for the rational design of functional luminescent and photocatalytic materials.

### 3.4 Electronic Structure

The DRS spectra (Figure S10) of the Eu-doped materials show a broad peak in the UV region between 220 and 370 nm. The wide band is composed of two main transitions: (i) host absorption (∼230 nm), and (ii) Eu – O charge transfer (∼245 nm). As for the Tb series, two distinct bands are observed: a sharp band around 230 nm, attributed to the host absorption, and a broad band between 260 and 370 nm, consistent with the Tb^3+^ f-d absorption.

The electronic properties of Eu^3+^- and Tb^3+^-doped Lu_2_O_3_ samples were investigated through Mott–Schottky (M–S) analysis, aiming to correlate the flat-band potential (V_fb_) with the band-edge positions derived from optical data (Figure S11). All potentials were measured versus the normal hydrogen electrode (NHE).^71^ The extracted parameters are summarized in Table 1.

**Table 1:** Calculated Electronic Flat Band (*V*_fb_), Charge Density, Band Gap (*E*_BG_), Valence and Conduction Band Energies (*E*_VB_, and *E*_CB_) of the Lu_2_O_3_:RE^3+^ (RE:Eu, or Tb) Materials

**Lu**_**2**_ **O**_**3**_ **:RE** ^**3+**^(*x* min)	***V***_**fb**_ (V)	**charge density** **(cm** ^**–3**^ **)**	***E***_**gap**_ (eV)	***E***_**VB**_ (eV)	***E***_**CB**_ (eV)
Eu (5)	–0.596	2.15 × 10^10^	5.60	–9.91	–4.31
Eu (10)	–0.665	2.11 × 10^10^	5.64	–10.02	–4.38
Eu (15)	–0.636	1.91 × 10^10^	5.62	–9.97	–4.35
Eu (20)	–0.616	2.08 × 10^10^	5.61	–9.94	–4.33
Tb (5)	–0.809	1.13 × 10^10^	5.58	–10.1	–4.52
Tb (10)	–0.688	1.81 × 10^10^	5.57	–9.97	–4.40
Tb (15)	–0.825	1.26 × 10^10^	5.60	–10.14	–4.54
Tb (20)	–0.675	1.16 × 10^10^	5.55	–9.94	–4.39

The flat-band potential ranged from approximately −0.60 to −0.83 V vs NHE, with the Eu-doped samples showing slightly less negative values than the Tb-doped counterparts. Charge carrier densities obtained from the M–S slopes were on the order of 10^10^ cm^–3^, confirming the highly insulating character of Lu_2_O_3_.^72^ The low donor concentration also implies a large depletion width, which can significantly affect the accuracy of the linear M–S fit and increase the influence of surface states.

The optical bandgaps, determined from diffuse reflectance (DRS) data, ranged from 5.55 to 5.64 eV, consistent with a wide-bandgap insulator. The valence and conduction band positions, calculated from the bandgap and valence band edge energies, are located at approximately +5.5 and 0.0 V vs NHE, respectively. These values indicate that the materials possess strong oxidative potential but only marginally reducing power, as the conduction band lies near the hydrogen evolution potential.

A systematic comparison between Eu^3+^- and Tb^3+^-doped samples revealed that Eu^3+^ incorporation tends to increase the apparent carrier density and shift V_fb_ to slightly less negative values, suggesting a higher density of shallow donor states or oxygen vacancies. In contrast, Tb^3+^-doped samples exhibited lower carrier densities and more negative flat-band potentials, consistent with greater charge compensation or deeper trap states. These effects may arise from differences in the electronic configurations of the dopant 4f levels and their interactions with Lu_2_O_3_ lattice defects.

Interestingly, the flat-band potentials determined electrochemically are about 0.5–0.9 V more negative than the calculated conduction band edges. Such discrepancies are often associated with interfacial effects, including surface states, Helmholtz-layer potentials, or frequency-dependent dispersion in the capacitance response. Therefore, the *V*_fb_ values should be interpreted as apparent flat-band potential rather than exact indicators of the conduction band minimum. Complementary measurements such as ultraviolet photoelectron spectroscopy (UPS) or X-ray photoelectron spectroscopy (XPS) would be necessary to confirm the absolute band alignment.^73–77^

Overall, these results confirm that both Eu^3+^ and Tb^3+^ doping only slightly affect the bulk band structure of Lu_2_O_3_, but significantly influence the electronic surface behavior, which may be crucial for applications involving charge transfer or photoactivation under UV excitation. When particle sizes are explicitly considered, the Mott–Schottky response of Lu_2_O_3_ nanoparticles reflects a transition from fully depleted, surface-dominated behavior at small sizes to mixed surface/space-charge effects near 50 nm, with dopant-dependent trends remaining robust across both regimes.

Using experimental data, a diagram of the positions of the Lu_2_O_3_ band gap energies was plotted, along with data on the formation of superoxide and hydroxyl radicals (Figure 7). The diagram indicates that the conduction band is well aligned with the formation of the superoxide radical. The result suggests that superoxide radicals can be formed if the material is excited in the band-to-band transition. To understand the role of the Eu and Tb ions on the electronic structure, the Dorenbos model is used.^78–80^ The position of the Eu^2+^ ground state was estimated by the charge transfer transition O^2–^ → Eu^3+^ experimentally obtained. To estimate the Tb^3+^ ground-state energy level, the field approximation U­(6, A) was set to 6.8 eV, as reported in the literature.^81–84^

**Figure 7:**
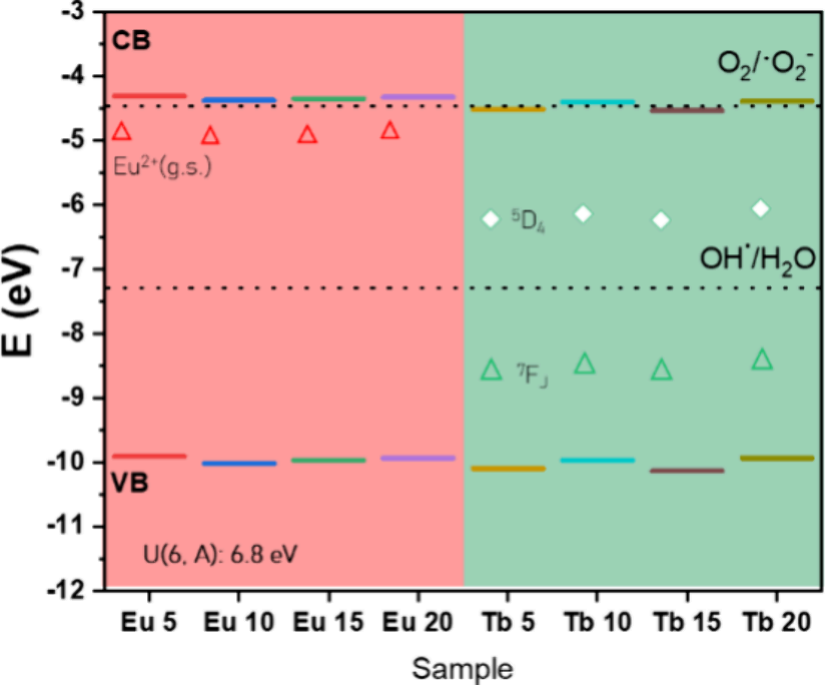
Energy diagram of the obtained materials compared with the reported values for radical formation. The conduction band energies were calculated using the flat band potential. The band gap energies were estimated using UV–vis spectroscopy. The HRBE positions were calculated using the Eu–O charge-transfer model.

The photocatalytic degradation of tetracycline was investigated under simulated solar irradiation using rare-earth-doped Lu_2_O_3_-based materials synthesized under different conditions (Figure 8a,b).

**Figure 8:**
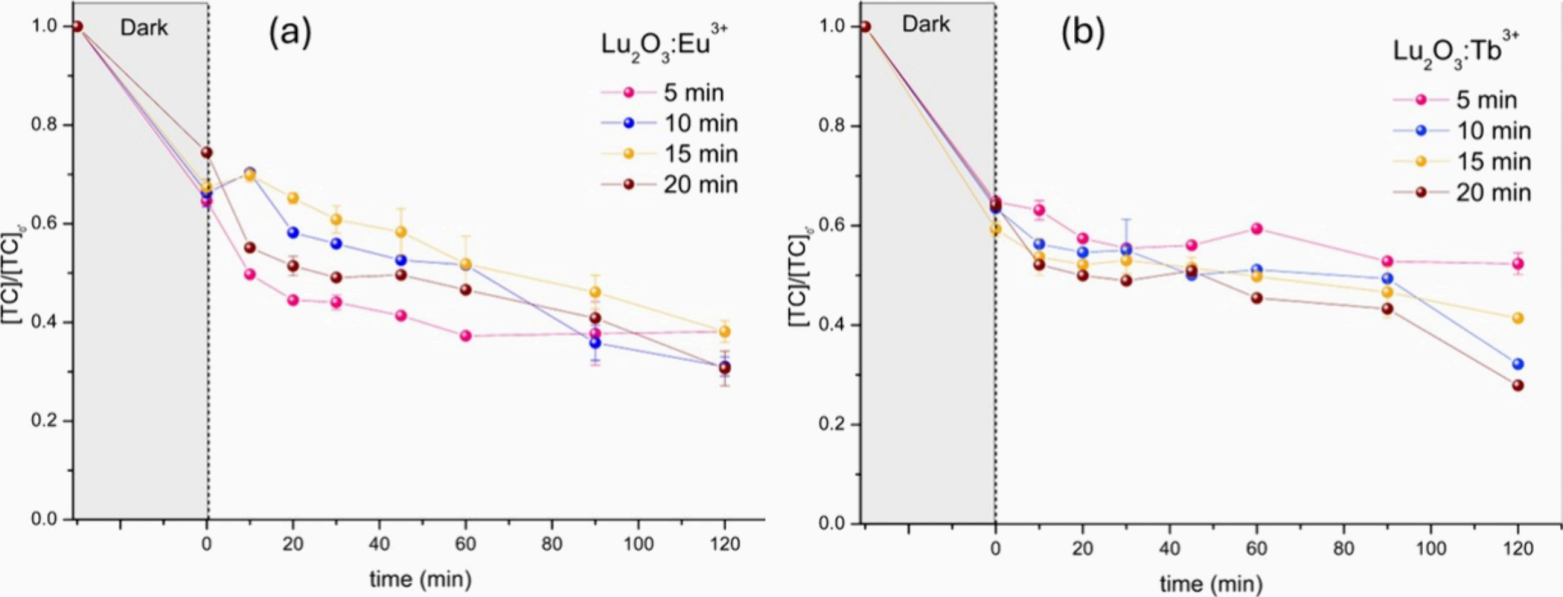
Solar lamp photodegradation of tetracycline antibiotics by the Lu_2_O_3_:Tb^3+^ (a) and Lu_2_O_3_:Eu^3+^ (b) nanoparticles obtained by different microwave irradiation times.

Among the evaluated samples, the Eu-doped materials exhibited the highest photocatalytic performance. In particular, the Eu-doped (10 min) sample achieved a final degradation efficiency of 53.1%, while the 20 min sample reached the highest overall degradation of 59.3%. For the Tb-doped series, the most efficient material was 20 min, a degradation efficiency of 56.5%. In contrast, the lowest activities were observed for Tb-doped (5 min; 47.7% efficiency) and Eu-doped (10 min; 57.6% efficiency), indicating that both dopant type and synthesis duration significantly influence photocatalytic behavior.

The photocatalytic performance of Lu_2_O_3_:RE^3+^ (RE = Eu, Tb) nanoparticles synthesized under microwave irradiation is best understood through the analysis of the apparent photocatalytic rate constants, *k*_app_, extracted from pseudo–first-order kinetic modeling (Table S4).^85^ These constants provide a quantitative measure of catalytic efficiency and enable direct correlation with structural and kinetic descriptors obtained from XRD and growth kinetics analyses.

The extracted *k*_app_ values exhibit a clear dependence on both synthesis time and dopant identity. Eu-doped Lu_2_O_3_ consistently displays higher apparent rate constants than Tb-doped samples synthesized under comparable conditions, confirming the superior photocatalytic activity of Eu-containing materials. For both dopants, *k*_app_ shows a nonmonotonic dependence on microwave irradiation time, reaching maximum values at intermediate synthesis times (10–15 min) before decreasing slightly at longer times. This behavior indicates that photocatalytic efficiency is not directly proportional to crystallite size, which increases monotonically with synthesis time, but instead reflects a balance between defect density, charge-carrier mobility, and recombination processes.

Correlation of *k*_app_ with structural parameters reveals that lattice microstrain is a stronger predictor of photocatalytic kinetics than crystallite size. Samples exhibiting intermediate microstrain values show the highest *k*_app_. In contrast, excessive microstrain at early synthesis times lead to enhanced charge recombination, and overly relaxed lattices at long synthesis times result in a reduced density of catalytically active defect sites. In particular, the *k*_app_ for the Eu^3+^-doped samples follows the exact monotonic trend of the asymmetry ratio and CT energies over the 10–20 min interval.

The relationships among photoluminescence, photocatalytic kinetics, and formation kinetics further emphasize the nonequilibrium nature of the system. The apparent photocatalytic rate constants scale with the Ostwald ripening rate constants determined from crystallite growth analysis, indicating that faster defect-mediated mass transport during synthesis results in defect configurations that enhance charge separation during photocatalysis. In this context, *k*_app_ can be regarded as a functional fingerprint of the kinetic growth pathway imposed by microwave irradiation.

Microstrain-induced lattice distortions and associated defect states are expected to generate local electric fields and trap distributions that modulate charge-carrier separation and recombination dynamics, providing a physical basis for the observed correlation between ε and photocatalytic activity.

These combined results demonstrate that photocatalytic kinetics in Lu_2_O_3_:RE^3+^ nanoparticles are governed by defect-mediated electronic structure effects encoded during nonequilibrium growth, rather than by equilibrium particle size or surface area considerations alone.

## 4 Conclusion

Eu^3+^- and Tb^3+^-doped Lu_2_O_3_ nanocrystals were rapidly synthesized by solvent-free microwave-assisted thermolysis, yielding phase-pure cubic nanoparticles within minutes. Time-resolved analysis shows that crystallite growth is governed mainly by diffusion-controlled Ostwald ripening, with Eu^3+^ producing higher ripening rates than Tb^3+^ due to stronger dopant-induced strain and defect-mediated mass transport. Williamson–Hall, photoluminescence, XPS, diffuse reflectance, and Mott–Schottky analyses identify microstrain as a key descriptor linking nonequilibrium growth to surface electronic structure and interfacial charge-transfer behavior, while the bulk band structure remains largely unchanged. Photocatalytic degradation of tetracycline further confirms that functional performance scales with microstrain and ripening kinetics rather than crystallite size alone. Together, these results establish a quantitative link between nonequilibrium synthesis, defect-mediated growth, and functionality in rare-earth sesquioxide nanocrystals.

## Supplementary Material



## Notes

Details on the microwave heating parameters, Scherrer and W–H analyses, kinetic modeling parameters, TEM particle size distribution, EDS, XPS, DRS, Mott–Schottky, and photocatalytic analysis (PDF)
