# Supplementary material for: Kinetically Encoded Microstrain Governs Growth, Electronic Structure, and Functionality in Microwave-Synthesized Lu2O3:RE3+ Nanoparticles
Source: ACS Omega. 2026 May 4;11(19):28834–46. doi: 10.1021/acsomega.6c01544 (PMC13191534; doi:10.1021/acsomega.6c01544)
Supplement: Supplementary file 1 [file ao6c01544_si_001.pdf]

Kinetically Encoded Microstrain Governs Growth, Electronic Structure, and Functionality in Microwave-Synthesized $Lu_2O_3:RE^{3+}$ Nanoparticles

*Felipe Ribeiro de Vasconcelos,[a] Priscila Hasse Palharim,[a] Caroline Helena Claudino,[a] Hermi Felinto Brito,[b] Juliana dos Santos de Souza,[a] José Miranda de Carvalho[a,]**

a. Federal University of ABC, Center for Natural and Humanities Sciences (CCNH), Avenida dos Estados, 5001, Santo Andre, São Paulo, Brazil, 09210-170

b. University of São Paulo, Institute of Chemistry, Av. Prof. Lineu Prestes, 748, São Paulo - SP, 05508-900, Brazil.

Summary

1. Microwave heating parameters …………………………………………………...3
2. Scherrer equation and crystallite size Calculation ……………………………..5
3. Kinetic Modeling ……………………………………………………………7
4. Williamson-Hall Analysis …………………………………………………10
5. Particle size distribution …………………………………………………16
6. EDS ……………………………………………………………………...17
7. XPS ……………………………………………………………………...19
8. DRS and Mott-Schottky …………………………………………………20
9. Photocatalytic Analysis …………………………………………………23

1. Microwave heating parameters

Microwave heating experiments were carried out using a domestic microwave oven (Electrolux MEO44) operating at 2.45 GHz with a rated output power of 1400 W and an internal cavity volume of 34 L. The microwave power was applied at the maximum setting, corresponding to continuous magnetron operation (100% duty cycle).

The Fe_3O_4 powder was placed in a refractory crucible positioned at off-center using a rotation plate to minimize field asymmetry effects. Aluminosilicate bricks were used as thermal insulation. Heating was conducted under ambient atmosphere.

The temperature was measured using an industrial optical pyrometer IMPAC - IP-1850. Emissivity values were adjusted from ε = 0.88 at 5 min to ε = 0.68 at 20 min to account for surface densification and phase evolution of iron oxides at high temperature. Error bars correspond to ±3% uncertainty.

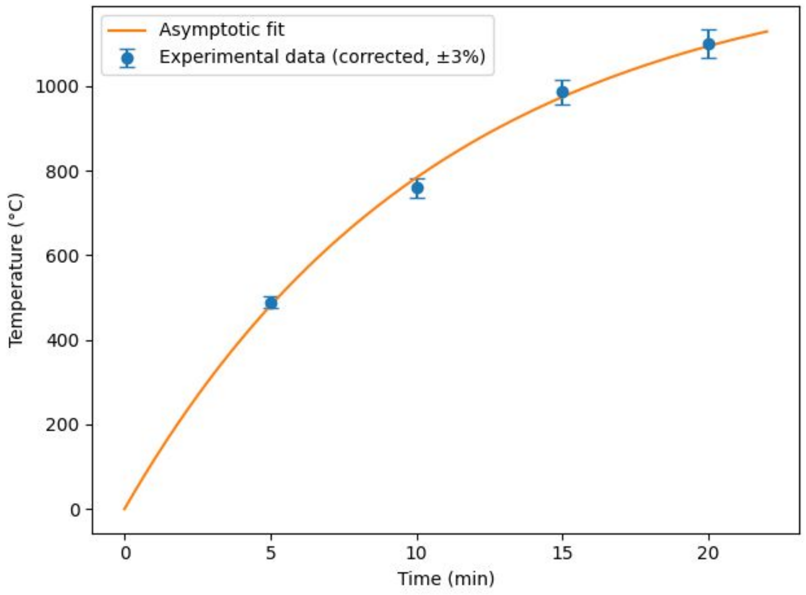

Figure S1. Microwave temperature measurements using an optical pyrometer. The heating was performed using the Fe_3O_4 susceptor alone.

Time (min)	ε used	T (°C)
5	0.88	480
10	0.82	733
15	0.72	920
20	0.68	1104

The emissivity-dynamic data was fitted using the asymptotic heating model:

$T(t) = T_{\max}(1 - e^{-kt})$ with:

- t in minutes
- T in °C

Uncertainties of ±3% were included for all points.

The temperature–time profile of Fe_3O_4 under microwave irradiation was well described by an asymptotic heating model, yielding an asymptotic temperature of 1265±70 °C and an effective heating constant of k_{heat} = 0.092±0.009 min^{-1}. Despite this formal asymptote, the system exhibits a practical thermal saturation near 1050–1100 °C within 20 min due to increasing radiative losses and reduced microwave absorption at high temperature.

2. Scherrer Equation and Crystallite Size Calculation

The Scherrer equation was applied to XRD peak widths to estimate coherent diffraction domain sizes (crystallite size):

$$D = \frac{K\lambda}{\beta_{corr} \cos\theta}$$

where:

- D is the crystallite size (Å or nm),

- K is the shape factor (assumed K = 0.9 for spherical particles),

- λ is the X-ray wavelength (Cu Kα, λ = 1.5406 Å),

- β is the full width at half maximum (FWHM) of the peak in radians (corrected for instrumental broadening),

- θ is the Bragg angle (in radians).

Instrumental broadening correction was applied as:

$$\beta_{corr}(2\theta) = \sqrt{\beta_{mean}^2 - \beta_{instr}^2}$$

Notes:

- $\beta_{instr}(2\theta)$ was taken as 0.003°, standard values for STOE STADI-P broadening parameter using the transmission method.

- Only peaks with relative intensity $I_{rel} > 0.3$ were used for the Scherrer analysis unless otherwise stated.

2.1 Peak detection and FWHM measurement

Peaks were detected using a local maxima approach after light smoothing of the intensity profile. FWHM values were measured by linear interpolation of the half-maximum points on the smoothed profile. Peaks with corrected $\beta_{corr} \leq 0$ were discarded (instrumental broadening larger than measured width).

2.2 Error estimation

The error in crystallite size for each sample was estimated as the standard deviation of D values computed from all selected peaks ($I_{rel} > 0.3$). This approach captures the spread of crystallite sizes arising from different reflections and provides an empirical uncertainty for the mean crystalite size reported.

3. Kinetic Modelling

Two classical kinetic models were fitted to the crystallite size data (mean D at each time point):

Ostwald ripening (diffusion-controlled):

$$D^3 = k \cdot t + {D_0}^3$$

Coalescence (collision/attachment-controlled):

$D^2 = k \cdot t + {D_0}^2$ Linear regression was applied to D^3 vs t and D^2 vs t to obtain k_{ripening} and $k_{\text{coalescence}}$, respectively. Goodness-of-fit was quantified using R^2, calculated from the observed and model-predicted D values.

3.1 Rate constant estimation (global and local)

Global zero-intercept rate constants

- Ostwald (global, intercept = 0): k_{ripening} (units: nm^3 / min)
- Coalescence (global, intercept = 0): $k_{\text{coalescence}}$ (units: nm^2 / min)

Finite differences between consecutive time points computed local (instantaneous) kinetic constants.

Local Rate Constant for Ostwald Ripening:

$$k_{ripening} \approx \frac{\Delta(D^3)}{\Delta t}$$

Local Rate Constant for Coalescence:

$$k_{coalescence} \approx \frac{\Delta(D^2)}{\Delta t}$$

These local values reveal temporal variations in growth rate and allow comparison between ripening and coalescence regimes.

Table S1. Local rate constant calculated for both ripening and coalescence models.

Material	Dopant	Kinetic model	Time interval (min)	Rate constant	Units	R^2	Type
Lu_2O_3	Eu^{3+}	Ostwald ripening	5–20	8.58(0.62) × 10^3	$nm^3 \cdot min^{-1}$	0.75	Global
Lu_2O_3	Tb^{3+}	Ostwald ripening	5–20	7.61(0.56) × 10^3	$nm^3 \cdot min^{-1}$	0.90	Global
Lu_2O_3	Eu^{3+}	Coalescence	5–20	1.61(0.30) × 10^2	$nm^2 \cdot min^{-1}$	0.72	Global
Lu_2O_3	Tb^{3+}	Coalescence	5–20	1.32(0.20) × 10^2	$nm^2 \cdot min^{-1}$	0.85	Global
Lu_2O_3	Eu^{3+}	Ostwald ripening	5–10	1.20(0.18) × 10^3	$nm^3 \cdot min^{-1}$	—	Local
Lu_2O_3	Eu^{3+}	Ostwald ripening	10–15	2.10(0.21) × 10^3	$nm^3 \cdot min^{-1}$	—	Local
Lu_2O_3	Eu^{3+}	Ostwald ripening	15–20	2.05(0.20) × 10^3	$nm^3 \cdot min^{-1}$	—	Local
Lu_2O_3	Tb^{3+}	Ostwald ripening	5–10	0.90(0.2) × 10^3	$nm^3 \cdot min^{-1}$	—	Local
Lu_2O_3	Tb^{3+}	Ostwald ripening	10–15	1.70(0.25) × 10^3	$nm^3 \cdot min^{-1}$	—	Local
Lu_2O_3	Tb^{3+}	Ostwald ripening	15–20	1.85(0.23) × 10^3	$nm^3 \cdot min^{-1}$	—	Local

Lu_2O_3	Eu^{3+}	Coalescence	5–10	2.20(0.13) × 10^2	$nm^2 \cdot min^{-1}$	—	Local
Lu_2O_3	Eu^{3+}	Coalescence	10–15	1.80(0.12) × 10^2	$nm^2 \cdot min^{-1}$	—	Local
Lu_2O_3	Eu^{3+}	Coalescence	15–20	1.40(0.09) × 10^2	$nm^2 \cdot min^{-1}$	—	Local
Lu_2O_3	Tb^{3+}	Coalescence	5–10	1.90(0.17) × 10^2	$nm^2 \cdot min^{-1}$	—	Local
Lu_2O_3	Tb^{3+}	Coalescence	10–15	1.55(0.14) × 10^2	$nm^2 \cdot min^{-1}$	—	Local
Lu_2O_3	Tb^{3+}	Coalescence	15–20	1.20)(0.11) × 10^2	$nm^2 \cdot min^{-1}$	—	Local

4. Williamson–Hall Analysis of Crystallite Size and Microstrain

The total peak broadening is assumed to be the linear sum of size and strain contributions, expressed as:

$\beta\cos\theta = \frac{K\lambda}{D} + 4\varepsilon\sin\theta$where:

- β is the corrected FWHM (radians),
- $\theta$is the Bragg angle,
- $K = 0.9$ is the Scherrer shape factor (spherical crystallites),
- $\lambda = 1.5406$Å (Cu Kα radiation),
- D is the volume-averaged crystallite size,
- ε is the lattice microstrain.

Linear regression of $\beta\cos\theta$ vs $4\sin\theta$ yields:

- **Intercept:** $\frac{K\lambda}{D}$
- **Slope:** 4ε

Microstrain values were extracted directly from the fitted slope as:

$\varepsilon = \frac{\text{slope}}{4}$**Validity of the Williamson–Hall Assumptions**

The W–H approach assumes:

1. Uniform elastic microstrain across crystallites,

2. Isotropic strain distribution,
3. Negligible peak shape asymmetry.

These assumptions are reasonably satisfied only after the completion of nucleation. Consequently, W–H results obtained at the earliest synthesis time (5 min) must be interpreted with caution, as rapid nucleation, defect gradients, and non-steady-state growth dominate this regime.

For this reason, microstrain values at synthesis times ≥10 min were considered physically representative of steady-state crystallite growth and were preferentially used for correlation with kinetic parameters.

Reproducibility and Consistency

All W–H analyses were performed using the same corrected peak broadening values as the Scherrer size calculations, ensuring methodological consistency across size, strain, and kinetic analyses. This integrated approach strengthens the reliability of the mechanistic interpretation presented in the main text.

Table S2. Extracted microstrain values calculated by the Williamson-Hall method.

Sample	5 min	10 min	15 min	20 min
Lu_2O_3:Eu^{3+}	~6.0×10^{-4}	~4.8×10^{-4}	~4.6×10^{-4}	~2.8×10^{-4}

**Lu_2O_3:Tb^{3+}**	~4.5×10^{-4}	~3.8×10^{-4}	~3.4×10^{-4}	~2.5×10^{-4}

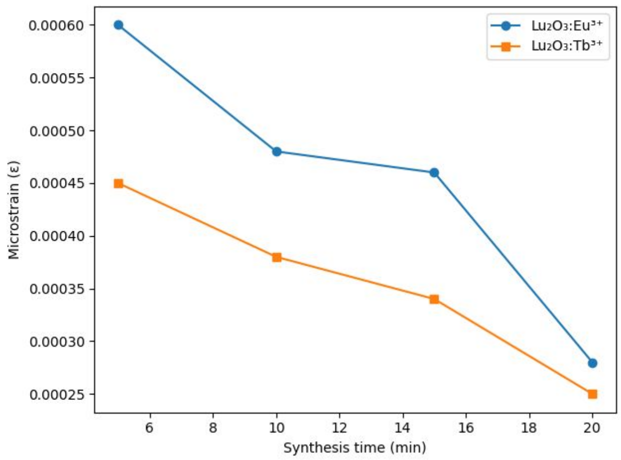

Figure S2. Temporal evolution of microstrain (ε) in Lu_2O_3:RE^{3+} (RE = Eu, Tb) nanoparticles extracted from Williamson–Hall analysis.

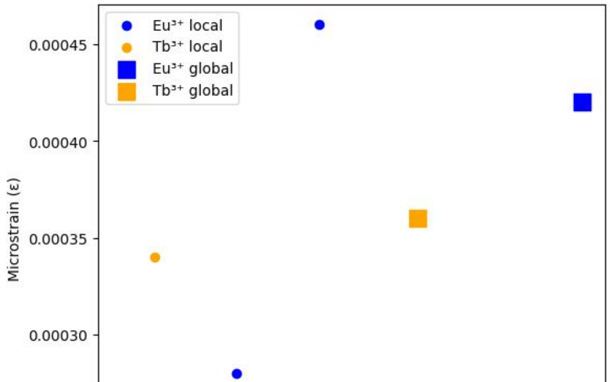

Figure S3. Correlation between microstrain (ε) obtained from Williamson–Hall analysis and Ostwald ripening rate constants (k_{OR}) for $Lu_2O_3:Eu^{3+}$ and $Lu_2O_3:Tb^{3+}$ nanoparticles. Local (circles) and global (squares) rate constants exhibit a positive trend, indicating that defect-induced microstrain governs diffusion-controlled crystallite growth.

Table S3. Lattice parameter extracted from X-ray diffraction for $Lu_2O_3:RE^{3+}$ obtained by different microwave irradiation times.

Material	Dopant	Time (min)	***a*** (Å)	σ (Å)	Reflections used

Lu_2O_3	Eu^{3+}	5	10.432	0.094	3
Lu_2O_3	Eu^{3+}	10	10.462	0.122	4
Lu_2O_3	Eu^{3+}	15	10.463	0.121	4
Lu_2O_3	Eu^{3+}	20	10.455	0.123	4
Lu_2O_3	Tb^{3+}	5	10.497	0.163	2
Lu_2O_3	Tb^{3+}	10	10.450	0.153	3
Lu_2O_3	Tb^{3+}	15	10.461	0.147	3
Lu_2O_3	Tb^{3+}	20	10.441	0.161	3

5. Particle size distribution.

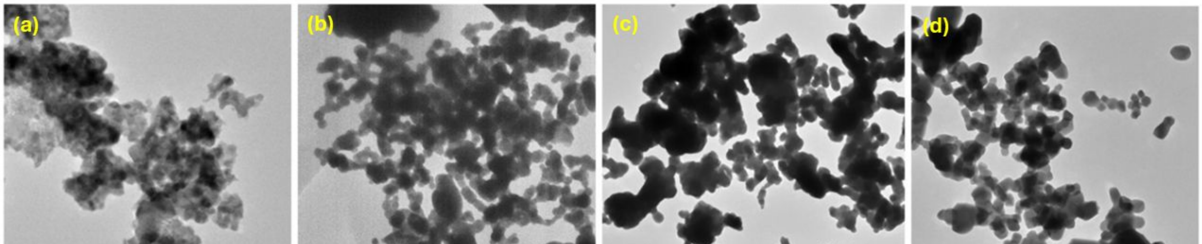

Figure S4. TEM images of the $Lu_2O_3:Tb^{3+}$ material obtained by different microwave irradiation time (5 min (a), 10 min (b), 15 min (c), and 20 min (d)). Particle size distribution of the $Lu_2O_3:Tb^{3+}$ materials obtained by microwave-assisted thermolysis with different irradiation times (e-h).

6. EDS

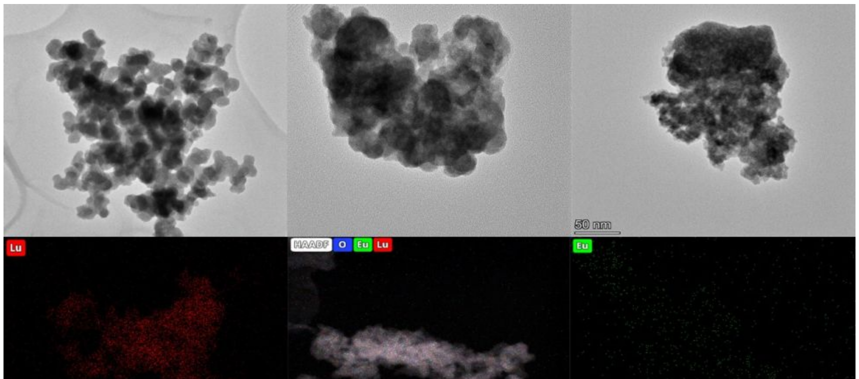

Figure S5. Transmission electron microscopy images and EDS elemental analysis of the $Lu_2O_3:Eu^{3+}$ materials after 5 minutes of microwave irradiation.

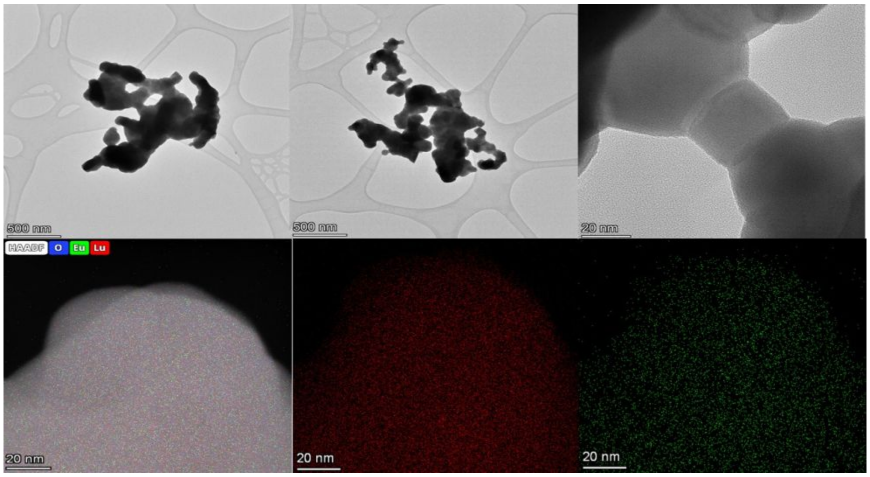

Figure S6. Transmission electron microscopy images and EDS elemental analysis of the $Lu_2O_3:Eu^{3+}$ materials after 20 minutes of microwave irradiation.

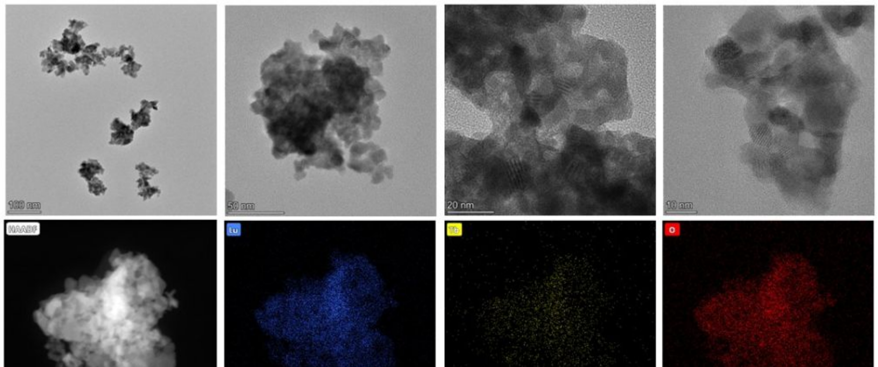

Figure S7. Transmission electron microscopy images and EDS elemental analysis of the Lu_2O_3:Tb^{3+} materials after 5 minutes of microwave irradiation.

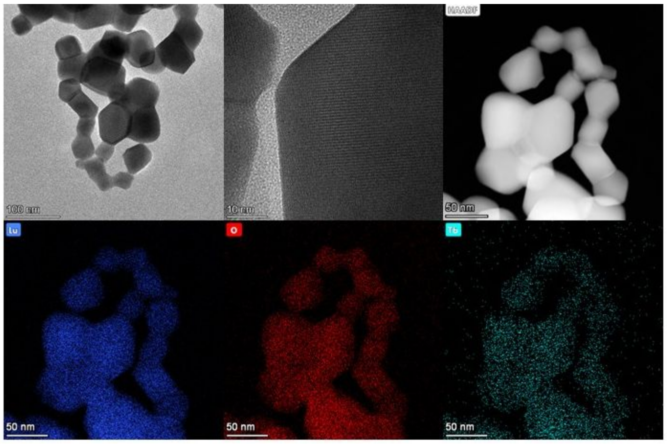

Figure S8. Transmission electron microscopy images and EDS elemental analysis of the Lu_2O_3:Tb^{3+} materials after 20 minutes of microwave irradiation.

7. XPS

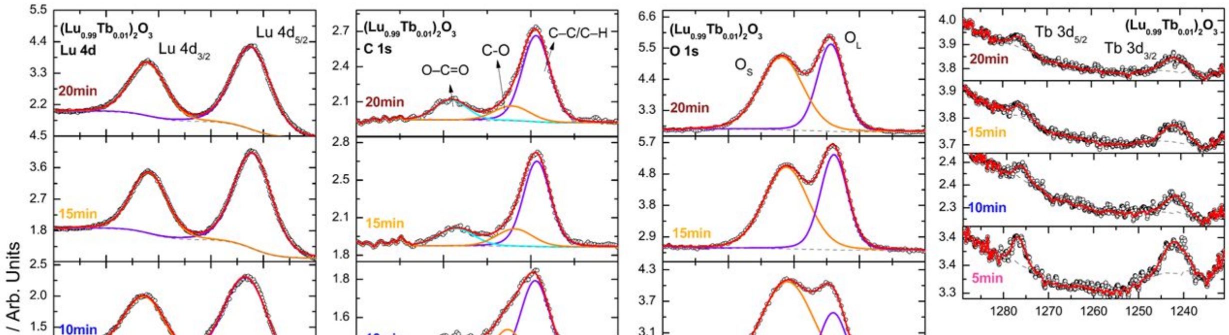

Figure S9. XPS analysis for the Tb-doped samples on the Lu (4d), C (1s), O (1s), and Tb (3d). Eu (3d) spectrum is also shown.

8. DRS and Mott-Schottky

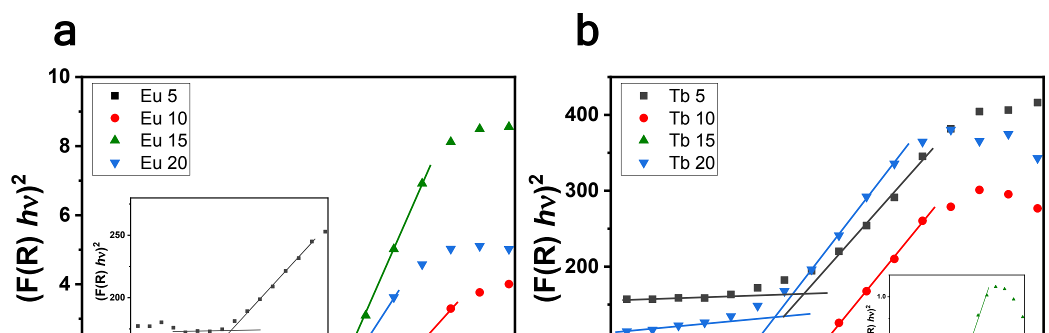

Figure S10. Diffuse reflectance spectra of $Lu_2O_3:RE^{3+}$ (RE: Eu (a), and Tb (b)) materials obtained with different microwave irradiation times.

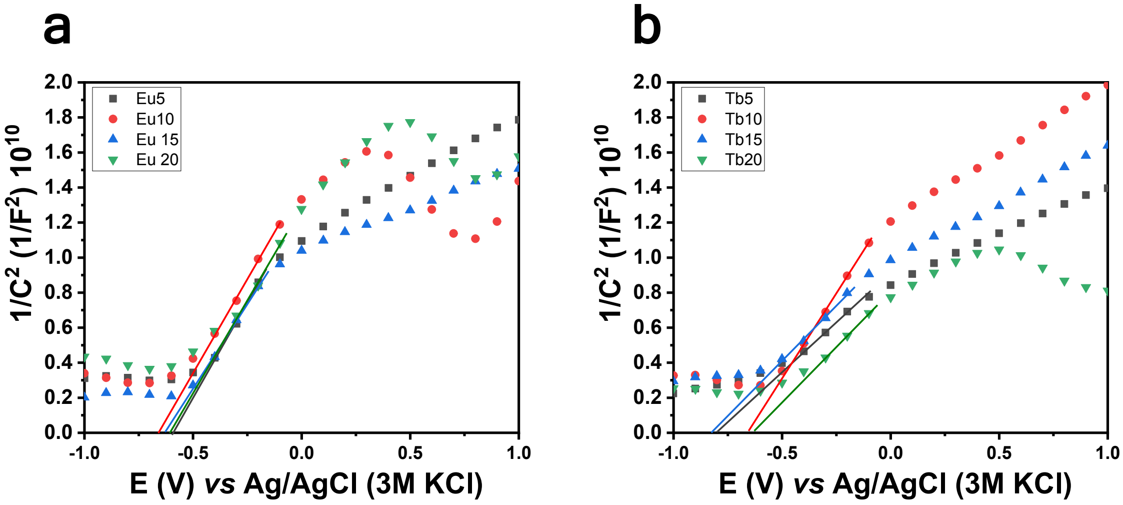

Figure S11. Mott-Schottky plots for the $Lu_2O_3:RE^{3+}$ (RE: Eu (a), and Tb (b)) materials obtained with different microwave irradiation times.

Band gap energy of the $Lu_2O_3:RE^{3+}$ films was estimated using the Kubelka-Munk Function from DRS data.

$$F(R) = K/S$$

where K is the molar absorption coefficient and S is the scattering factor:

$$K = (1 - R)^2$$

$$S = 2R$$

R is the reflectance of the material:

$$R = \%R/100$$

The combination leads to:

$$F(R) = (1 - R)^2/2R$$

To compare the energy levels of the Lu_2O_3:RE^{3+} materials with the potential of photoexcited radical formation, Mott-Schottky plots were taken using the relation as follows:

$$\frac{1}{C^2} = \left(\frac{2}{e\varepsilon\varepsilon_0 N_d}\right)\left[V_a - V_{fb} - \frac{kT}{e}\right]$$

where C is the space charge layers capacitance, e is the electron charge, ε is the dielectric constant, ε_0 is the permittivity of vacuum, N_d is the charge carrier density, V_a is the applied potential, and V_{fb} is the flat band potential. The flat band potential V_{fb} was determined by taking the x-intercept of a linear fit to the Mott-Schottky plot, $\frac{1}{C^2}$, as a function of applied potential (V_a) when the frequency is 1000 Hz,

The charge carriers' density N_d can be derived from the following equation:

$$N_d = \frac{2}{\varepsilon\varepsilon_0 e}\frac{dE}{d\frac{1}{C^2}} = \frac{2}{\varepsilon\varepsilon_0 e}\frac{1}{slope}$$

Both DRS and Mott-Schottky plots were combined to estimate the band structure of the materials. Assuming that the gap between flat band potential and the bottom edge of the conduction band is negligible for n-type semiconductors, the conduction band position of the bare catalysts could be estimated. The measurements were performed in pH 8.8. For converting the obtained potential (vs Ag/AgCl) to RHE (Reversible Hydrogen Electrode at pH = 0), the equation below was used:

$$E_{RHE} = E_{AgCl} + 0.059pH + E^{0}_{AgCl}$$

where, E^{0}_{AgCl} is 0.209 V. The E_{RHE} can be converted to energy in eV *vs.* vacuum level sing the Equation:

$$E_{VAC} = E_{RHE} - 4.44$$

9. Photocatalytic Kinetics Analysis

Photocatalytic Experiments and Data Acquisition

The photocatalytic measurements were performed under identical conditions for all samples, ensuring that differences in activity arise from intrinsic material properties rather than experimental variability.

Kinetic Model and Assumptions

The photocatalytic degradation kinetics were analyzed using a pseudo–first-order model, which is commonly applicable when the pollutant concentration is low and surface-active sites are in excess. The model is expressed as:

$\ln\left(\frac{C_0}{C}\right) = k_{\mathrm{app}}\, t$where:

- $C_0$is the initial pollutant concentration,
- C is the concentration at irradiation time t,
- k_{app} is the apparent first-order rate constant (min^{-1}).

This formalism assumes that the overall reaction rate is limited by the generation, separation, and transfer of photogenerated charge carriers rather than by mass transport in solution.

Data Treatment and Regression Procedure

To minimize the influence of lamp stabilization and residual adsorption effects, the initial irradiation interval (0–10 min) was excluded from the kinetic fitting. Linear

regressions of ln (C_0/C)versus time were performed over the irradiation window from 10 to 120 min for each sample.

The apparent rate constant $k_{\mathrm{app}}$was extracted as the slope of the linear fit. The linearity observed over the selected time window confirms the validity of the pseudo–first-order approximation for the present system.

Table S4. Apparent photocatalytic rate constants and fitting quality parameters.

Lu_2O_3:Tb^{3+} (min)	k_{app}(min^{-1})	R^2	RMS residual
**5**	1.29×10^{-3}	0.92	0.031
**10**	4.22×10^{-3}	0.97	0.018
**15**	2.40×10^{-3}	0.95	0.024
**20**	4.89×10^{-3}	0.98	0.015
**Lu_2O_3:Eu^{3+} (min)**	**k_{app}(min^{-1})**	**R^2**	**RMS residual**
**5**	2.27×10^{-3}	0.94	0.027
**10**	6.74×10^{-3}	0.98	0.014
**15**	5.47×10^{-3}	0.97	0.017
**20**	4.79×10^{-3}	0.99	0.012

Statistical assessment of structural descriptors (ANOVA analysis)

To statistically evaluate the relative contributions of structural descriptors to the observed kinetic, optical, and photocatalytic trends, an analysis of variance (ANOVA) was performed using lattice parameter, particle size (Scherrer), lattice microstrain (ε), and dopant identity (Eu^{3+} vs Tb^{3+}) as independent factors. The analysis indicates that variations in the lattice parameter do not contribute significantly to the variance in photocatalytic rate constants or growth kinetics ($p \gg 0.05$), consistent with the small magnitude of lattice expansion/contraction relative to instrumental uncertainty. Particle size exhibits a moderate effect; however, its statistical significance is strongly reduced when microstrain is included in the model, indicating that size acts as a correlated rather than primary descriptor. In contrast, microstrain emerges as a statistically significant factor ($p < 0.05$) with a large effect size, retaining explanatory power across kinetic, optical, and functional datasets. Furthermore, a significant interaction between microstrain and dopant identity is observed, rationalizing the monotonic ε–k_{app} relationship for Eu^{3+}-doped samples and its absence in Tb^{3+}-doped analogues. These results support the conclusion that kinetically encoded lattice microstrain, rather than lattice parameter variations or particle size alone, governs the structure–property relationships in the present non-equilibrium system.
